# Molecular Signaling Interactions and Transport at the Osteochondral Interface: A Review

**DOI:** 10.3389/fcell.2020.00750

**Published:** 2020-08-19

**Authors:** Mateus Oliveira Silva, Julia L. Gregory, Niloufar Ansari, Kathryn S. Stok

**Affiliations:** Department of Biomedical Engineering, University of Melbourne, Parkville, VIC, Australia

**Keywords:** joint, articular cartilage, bone, molecular signaling, transport, osteoarthritis

## Abstract

Articular joints are comprised of different tissues, including cartilage and bone, with distinctive structural and mechanical properties. Joint homeostasis depends on mechanical and biological integrity of these components and signaling exchanges between them. Chondrocytes and osteocytes actively sense, integrate, and convert mechanical forces into biochemical signals in cartilage and bone, respectively. The osteochondral interface between the bone and cartilage allows these tissues to communicate with each other and exchange signaling and nutritional molecules, and by that ensure an integrated response to mechanical stimuli. It is currently not well known how molecules are transported between these tissues. Measuring molecular transport *in vivo* is highly desirable for tracking cartilage degeneration and osteoarthritis progression. Since transport of contrast agents, which are used for joint imaging, also depend on diffusion through the cartilage extracellular matrix, contrast agent enhanced imaging may provide a high resolution, non-invasive method for investigating molecular transport in the osteochondral unit. Only a few techniques have been developed to track molecular transport at the osteochondral interface, and there appear opportunities for development in this field. This review will describe current knowledge of the molecular interactions and transport in the osteochondral interface and discuss the potential of using contrast agents for investigating molecular transport and structural changes of the joint.

## Introduction

Within a lifetime joints can undergo changes and progressive degeneration as a result of natural ageing or injury. These are the primary risk factors for the development of osteoarthritis (OA), a painful and debilitating condition affecting millions worldwide (Felson et al., 2013; Sanchez-Adams et al., 2014). The joint is a complex structure that relies on mechanical and biological integrity to function properly. To investigate these events, mechanobiology has emerged as a field that can address the dynamic interactions between cells and their mechanical environment. Understanding the complex interactions of the joint tissues, as well as the cellular, biochemical and mechanical responses at work, may provide insights into joint degeneration and OA (Lepage et al., 2019).

The joint is comprised largely of bone and cartilage, separated by an osteochondral interface that is comprised of deep layers of cartilage and the underlying subchondral bone. These individual components interact cooperatively to make a complex functional unit (Yuan et al., 2014). Due to the proximity of the joint layers, homeostasis is maintained through tightly regulated mechanoregulatory pathways that facilitate communication between tissues and responses to the environment (Li et al., 2013). Among the signaling molecules involved, transforming growth factor-β (TGF-β) and the protein Wnt, are key components in the development, growth, maintenance and repair of cartilage. Alterations in these pathways contribute to OA progression (Finnson et al., 2012).

In response to mechanical or biological stimuli, increased vascular activity and angiogenesis occurs in the subchondral bone. This process is largely mediated by vascular endothelial growth factor (VEGF), expressed by multiple sources within the joint. During OA, new blood vessels invade the deep layers of articular cartilage creating porous channels between the tissues. The newly formed channels increase the capacity for transport across the bone-cartilage interface, suggesting a direct path for the migration of biological factors and nutrients, thus enhancing the overall crosstalk and molecular interactions at the osteochondral interface (Greenwald and Haynes, 1969; Lane et al., 1977). Despite evidence for increased transport via vascular channels from the subchondral bone, the primary route for nutrients to access the extracellular matrix (ECM) and chondrocytes of the articular cartilage is via diffusion (Pan et al., 2009; Sharma et al., 2013; Villalvilla et al., 2013).

Both molecular size and mechanical loading affect diffusion transport rates at the osteochondral interface (Malinin and Ouellette, 2000; Sophia Fox et al., 2009; Di Luca et al., 2015). There is evidence to suggest an increase in molecular transport in OA, however, a lack of understanding of how materials transport to and from the tissues still remains, limiting our ability to develop new treatments for OA (Yuan et al., 2014). Since contrast agents may also be transported via diffusion through the cartilage ECM, contrast agent enhanced imaging modalities such as fluorescence microscopy, magnetic resonance imaging or X-ray computed tomography (CT), may serve as efficient, non-destructive techniques for investigating molecular transport at the osteochondral interface (Joshi et al., 2009; Kulmala et al., 2010; Choi and Gold, 2011; DiDomenico et al., 2018; Pouran et al., 2018). To date, few studies using contrast agent-based imaging have been performed to investigate solute transport through articular cartilage (Kulmala et al., 2010; Silvast et al., 2013; Arbabi et al., 2015; Kokkonen et al., 2017).

This review describes the structure and biological properties of the osteochondral interface, providing background for the reader. Followed by current knowledge on molecular interactions and transport at the osteochondral interface. Finally, we seek to highlight methods for investigating molecular transport, specifically with a view to understanding transport across the osteochondral interface and changes induced by disease progression.

## The Osteochondral Interface Is a Key Structure in Joint Pathophysiology

**FIGURE 1:**
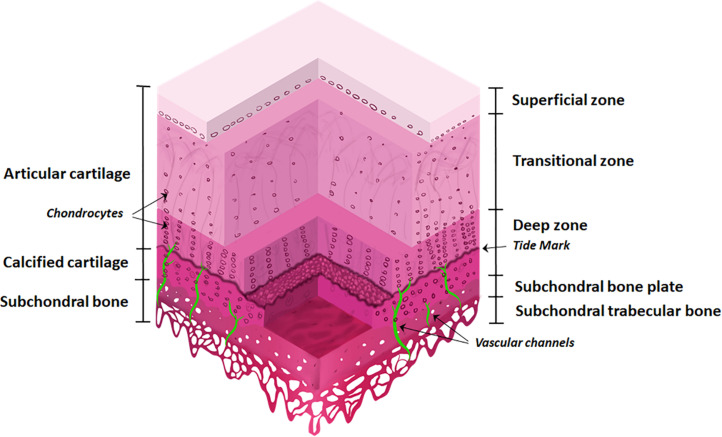
The osteochondral interface and bone and cartilage zones. The osteochondral interface lies between the connecting layers of articular cartilage and subchondral bone. The articular cartilage consists of three zones: superficial zone, transitional zone, and deep zone and finishes at the tidemark. Below this lies the calcified cartilage layer. The subchondral bone is located below the calcified cartilage and consists of the subchondral bone plate and subchondral trabecular bone. Vascular channels lie within the subchondral bone.

The osteochondral interface is a gradient tissue that consists of articular cartilage ∼90%, calcified cartilage ∼5%, and subchondral bone ∼5% (Figure 1). These tissues present different structural, mechanical and biological properties allowing the individual components to interact cooperatively forming an integrated functional unit (Yuan et al., 2014; Longley et al., 2018).

The articular cartilage consists of three zones, beginning at the joint space and finishing at the tidemark: (1) superficial zone (at the articular surface); (2) transition/middle zone, and (3) radial/deep zone (Sophia Fox et al., 2009; Suri and Walsh, 2012). This is followed by the tidemark and adjoining calcified cartilage layer. The subchondral bone layer lies at the bottom and includes the subchondral bone plate (Figure 1).

Articular cartilage is an avascular and alymphatic tissue, composed of ECM and embedded chondrocytes. Chondrocytes constitute only 2% of the total volume in cartilage and are responsible for the synthesis of the ECM (Hunziker et al., 2002). As they are the sole cell type present, chondrocytes are essential for maintaining cartilage integrity, by responding to growth factors, mechanical loads, and other physicochemical stimuli (Muir, 1995; Akkiraju and Nohe, 2015).

The ECM is comprised largely of collagen type II and proteoglycans. These components retain water within the cartilage, providing strength and stabilization to the tissue (Sophia Fox et al., 2009). The composition of ECM, its water content, and cell density vary in the different layers. The superficial zone makes up ∼10–20% of articular cartilage thickness, has the highest density of chondrocytes, and collagen fibrils that are tightly packed and aligned parallel to the articular surface. It also has the highest water content (∼80%) and solute transport compared to other zones (Eyre, 2002; Akkiraju and Nohe, 2015).

The transition zone makes up ∼40–60% of cartilage thickness. This layer has a low density of chondrocytes with cells spherical in shape. The collagen fibers are thick and have an oblique orientation (Sophia Fox et al., 2009).

The deep zone makes up ∼30% of cartilage thickness. This layer has the highest level of proteoglycans and the lowest water content (∼60%). The chondrocytes are aligned in typical columns, perpendicular to the articular surface. Collagen fibers are thick and orientated parallel to the cell columns (Sophia Fox et al., 2009).

The layer that interfaces with the subchondral bone is the calcified cartilage, anchoring the articular cartilage to the bone with collagen fibers of the cartilage deep zone (Martel-Pelletier et al., 2008). The calcified layer is split into zones by the tidemark and presents characteristics of both cartilage (the deposition of collagen type X) and bone (presence of alkaline phosphatase and mineral deposits). It contains a very low density of hydrophobic chondrocytes (Martel-Pelletier et al., 2008).

The subchondral bone is comprised of both inorganic and organic components. Crystalline hydroxyapatite is the most abundant inorganic component of the bone matrix, while calcium, carbonate, phosphate and other inorganic elements are also present at low levels (Feng, 2009). The organic components are collagen type I fibers (90%), and proteins including enzymes, cytokines, and growth factors. The subchondral bone contains vessels and channels which are essential for supplying nutrients to the bone and potentially the deeper layers of cartilage, thus also providing transport pathways for signaling molecules and factors across the osteochondral interface (Robling et al., 2006; Li et al., 2013).

**FIGURE 2:**
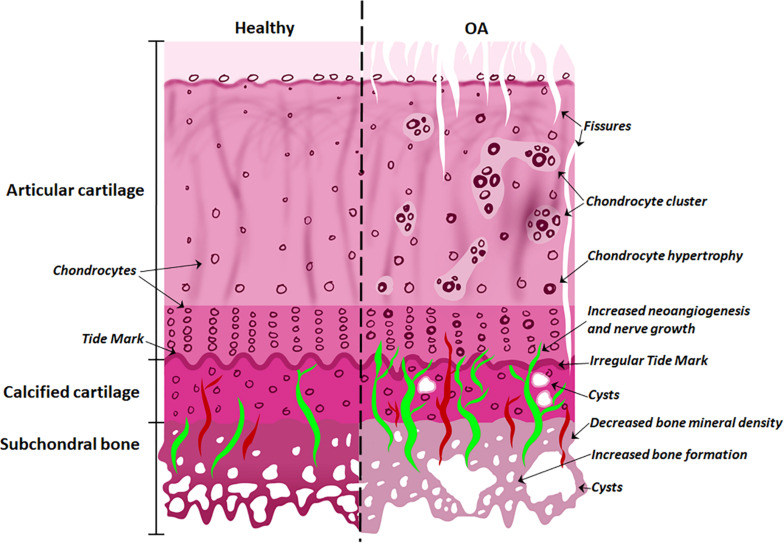
Differences between normal and osteoarthritic osteochondral tissues. The features of the healthy osteochondral interface are homogeneous articular surface, normal chondrocytes and vascularization of the subchondral bone. With the onset of OA, the articular surface exhibits fissures; chondrocyte hypertrophy and clusters; while cysts appear in the calcified cartilage; with increased angiogenesis and nerve growth penetrating the tide mark; and decreased bone mineral density and increased bone formation in the subchondral bone.

With the onset of OA (Figure 2), the subchondral bone plate begins to thicken and perforate. The bone is a poor-quality (low mineral) bone, the trabecular architecture alters, and bone cysts appear. In the cartilage, there is a breakdown in the collagen network, leading to swelling and eventual reduction in proteoglycan content, as well as fissures and cracks across the osteochondral interface. The tidemark becomes irregular. Additionally, bone remodeling stimulates new vascularization and nerve growth from already formed blood vessels and nerves in the subchondral bone (Goldring and Goldring, 2016; Lepage et al., 2019). These structural alterations are tightly linked to molecular signaling interactions at the osteochondral interface.

## Molecular Signaling Interactions at the Osteochondral Interface

Among the diverse mechanoregulatory pathways involved in joint pathophysiology, TGF-β and Wnt play an integral role in both the maintenance and degradation of cartilage via signaling across the osteochondral interface. Specifically, deregulation of TGF-β and Wnt signaling causes instability in ECM structure and function, and alters chondrocyte development, contributing to OA progression (Finnson et al., 2012).

### TGF-β

**FIGURE 3:**
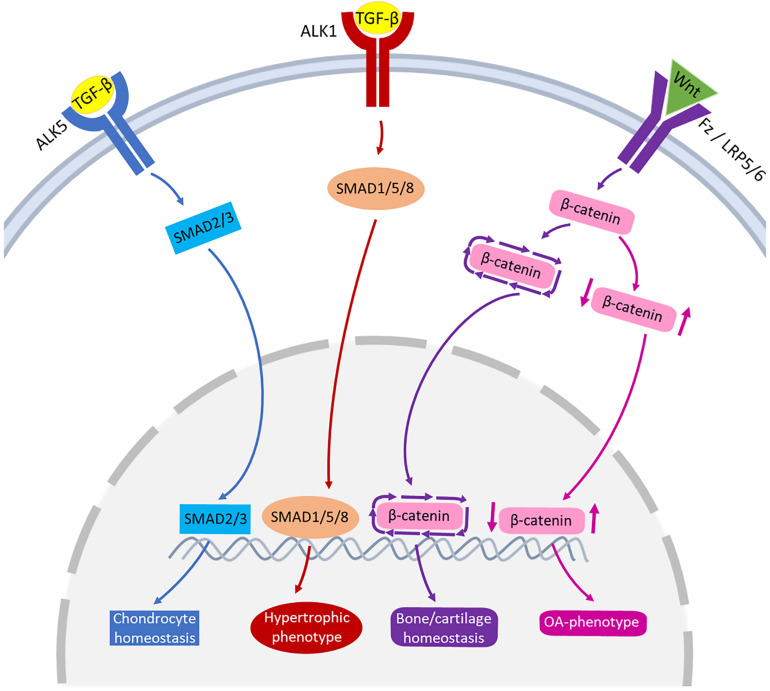
Schematic representation of the signaling pathways of TGF-β and Wnt involved in joint pathophysiology. TGF-β can bind to ALK5, that activates SMAD2/3 leading to chondrocyte homeostasis (blue pathway arrows). TGF-β can also bind to ALK1, activating SMAD1/5/8 that may lead to hypertrophic phenotype of chondrocytes (red pathway arrows). Wnt binds to receptors Fz or LRP5/6 activating β-catenin synthesis. A healthy expression of β-catenin leads to bone/cartilage homeostasis (purple pathway arrows) while imbalanced β-catenin can result in an OA-phenotype (pink pathway arrows).

The TGF-β family are multipurpose growth factors that play a fundamental role in cartilage development, homeostasis and repair. They consist of up to 35 members including TGF-βs, bone morphogenetic proteins (BMPs), growth and differentiation factors (GDFs) and activins (Finnson et al., 2012; Thielen et al., 2019). To initiate signaling (Figure 3), growth factors bind to membrane-bound activin-like kinase receptors (ALK5 or ALK1), in turn activating SMAD2/SMAD3 phosphorylation or SMAD1/5/8 signaling pathways, respectively. The availability of TGF-β determines which signaling pathway is activated (Remst et al., 2014; van der Kraan, 2017). During regular physiological loading of healthy joints, readily available TGF-β will signal via the ALK5-SMAD2/3 pathway, thus driving a protective role for cartilage by maintaining chondrocyte metabolism and survival. In a pathologic setting such as OA or in aging, the role of TGF-β may shift and the ALK1-SMAD1/5/8 pathway dominates. This causes a hypertrophic phenotype in chondrocytes, resulting in an imbalance in ECM turnover (Retting et al., 2009; Li et al., 2010; Chen et al., 2012). The TGF-β/SMAD signaling pathways are essential for maintaining cartilage integrity and importantly, chondrocyte function.

The importance of TGF-β in OA has been well established in numerous studies to date. Animal models with genetic alterations in signaling molecules of the TGF-β pathway including *Smad* gene mutations, ALK5 knock-outs, and overexpression of TGF-β receptor II develop features of OA, including cartilage damage and alterations in chondrocyte differentiation (Serra et al., 1997; Yang et al., 2001; Blaney Davidson et al., 2006; Shen et al., 2013; Wang et al., 2017). Mice with modifications to the *Smad3* gene show a loss of articular cartilage, reduced proteoglycans and increased chondrocyte differentiation (Yang et al., 2001). Conditional knockout mice for TGF-β receptor II in chondrocytes specifically, developed a severe form of OA-like disease with hypertrophic chondrocytes and degraded cartilage (Shen et al., 2013).

TGF-β signaling also contributes to cartilage maintenance and integrity by controlling inflammatory cytokine production. The proinflammatory cytokines, IL-1β and TNF-α are produced by multiple sources in joint tissue including chondrocytes (Wojdasiewicz et al., 2014). They are potent inducers of matrix metalloproteinases (MMPs) that are responsible for cleaving ECM components to maintain normal matrix remodeling and excessive degradation of cartilage during OA (Vincenti and Brinckerhoff, 2001; Burrage et al., 2006; Wojdasiewicz et al., 2014). The proteases MMP-1 and MMP-13 are among the most prevalent in OA cartilage and target collagens I, II, and III, thus contributing directly to disease progression (Mitchell et al., 1996; Vincenti et al., 1998; Martel-Pelletier et al., 2008). Another key activator of MMPs in OA tissue is the Wnt glycoproteins. The Wnt signaling pathway has a key role in maintaining cartilage and bone homeostasis (Zhou et al., 2017).

### Wnt Signaling

One of the common pathways that Wnt molecules use to drive their downstream transcriptional events is the canonical signaling pathway (Figure 3). Wnt binds to its receptors, Frizzled (Fz) and low-density lipoprotein receptor-related protein 5/6 (LRP5/6), activating β-catenin synthesis which in turn accumulates in the cell nucleus for use in gene transcription. In the absence of Wnt, β-catenin is degraded and prevented from translocating to the cell nucleus (Krause and Gregory, 2012; Li et al., 2012). There is a fine balance between the level of Wnt-Fz activation and the accumulation of β-catenin in cells that can escalate to an OA-like phenotype. Both excessive and insufficient Wnt activation, as well as increased and reduced β-catenin signaling, can result in cartilage degradation, chondrocyte apoptosis, subchondral bone damage and osteophyte formation (Lories et al., 2007; Zhu et al., 2008, 2009; Zhou et al., 2017).

Mice with a chondrocyte-specific transgene for β-catenin and T cell factor (ICAT) have reduced β-catenin signaling that causes significant articular cartilage damage and high levels of chondrocyte apoptosis (Zhu et al., 2008). Similarly, preventing the natural degradation of β-catenin (which occurs in the absence of Wnt-Fz activation) by inhibiting the kinase, GSK3β, results in reduced β-catenin signaling (Miclea et al., 2011). Mouse cultured metatarsal explants with the GSK3β inhibitor had reduced chondrocyte proliferation, loss of proteoglycans and cartilage degradation. Microarray analysis showed increased MMP expression and down-regulation of cartilage ECM proteins (Miclea et al., 2011). On the contrary, transgenic mice with conditional β-catenin activation have an increase in β-catenin levels in articular chondrocytes. This causes an overall accelerated OA-like phenotype with increased rates of chondrocyte development (Zhu et al., 2009).

## Transport at the Osteochondral Interface

The presence of vascular networks in the subchondral bone and, to a lesser extent, the calcified cartilage regions of normal healthy joints has been well established (Bullough and Jagannath, 1983). Early evidence for these transport pathways was observed using electron microscopy in healthy tissue. This included the appearance of holes with vessel-like features in the subchondral bone of human tibias (Duncan et al., 1987). Studies done in the joints of rabbits and canines further supported these observations showing small vascular canals and capillaries running through the subchondral bone region. Interestingly, the majority of these structures were not present in calcified or deeper layers of the cartilage (Clark, 1990).

In contrast, during the early stages of OA (Botter et al., 2011; Goldring and Goldring, 2016), bone remodeling stimulates new vascularization from already formed blood vessels in the subchondral bone (Figure 2). Histological evidence in human knee cartilage has shown porous channel formation and accompanying blood vessels that pass through the calcified cartilage, across the tidemark and into the deep zone of articular cartilage (Suri et al., 2007; Walsh et al., 2007). Models of OA in both rabbits and rats as well as human clinical samples have also shown a positive correlation between increasing angiogenic activity and increased vascular invasion in articular cartilage in the early stages of disease (Franses et al., 2010; Ashraf et al., 2011; Saito et al., 2012; Li et al., 2013).

Despite the evidence for transport across the osteochondral interface via *de novo* vasculature formation penetrating the cartilage tissue during OA, there is currently no functional evidence to support this route of transfer, and thus diffusion of solutes and nutrients is considered the primary method of transport.

Solute transport through the ECM is crucial to chondrocyte physiology and maintenance of biochemical and mechanical integrity (Evans and Quinn, 2005). Solute physicochemical properties (size, shape, charge, and concentration), mechanical environment (such as loading or unloading), and tissue properties (composition and structure) are all factors that contribute to effective molecular transport at the osteochondral interface (Knothe Tate et al., 1998; Knothe Tate and Knothe, 2000; Farnum et al., 2006; Serrat et al., 2009, 2014; Knothe Tate et al., 2012; Ngo et al., 2018). Changes in tissue structures during OA would affect solute diffusion kinetics in the osteochondral interface due to altered subchondral bone porosity, tide mark perforations and cartilage permeability (Figure 2).

### Solute Physicochemical Effect on Molecular Transport

Molecular size is one of the most important factors determining the nature and rate of transport, affecting the interaction of the molecule and the pores in the cartilage. There is an inverse relationship between the molecular size and its diffusivity (also called mass diffusivity or diffusion coefficient) (Leddy and Guilak, 2003). Diffusivity is the rate of material transport; i.e., quantity of a substance fluxing through a surface per unit of time. Molecular size also has an inverse correlation to partition coefficient, which is the ratio of the concentrations of a solute in two layers after equilibrium (Kokkonen et al., 2017).

Since different layers of articular cartilage have different structures, diffusivity of a single molecule varies as it passes through these zones. An *ex vivo* study of the transport kinetics of different size dextrans showed that small and large dextrans (with radii of ∼2 and 15 nm, respectively) have their highest diffusivity in the superficial zone, however, surprisingly, the middle sizes (with radii of ∼6–7 nm) had the highest diffusivity in the middle and deep zones (Shoga et al., 2017).

In contrast to cartilage, bone tissue has both vascular networks and lacunar-canalicular systems that enable transport of nutrients from blood circulation to osteocytes. To affect osteocytes, it is critical for macromolecules to be able to pass through the canaliculi to reach the cells. In humans, the average canalicular diameter has been shown to be ∼315 nm, while the diameter of the osteocyte dendritic process is ∼145 nm, leaving a pericellular space surrounding each process of ∼85 nm (Dallas et al., 2013). Murine samples with an average ∼260 nm canalicular diameter and ∼100 nm osteocyte dendritic process, have a similar pericellular space (∼80 nm), suggesting that molecules larger than this size cannot pass through the lacunar-canalicular system (Wang, 2018).

Studies have shown that the rate of transport depends on both size and shape of solute (summarized in Table 1). This was assessed by using fluorescent tracers between ∼375 and 43,000 Da in size and a range of shapes (linear, spherical and globular). Transport of larger and linear molecules in bone, was slower than smaller molecules of the same shape and also globular molecules of similar molecular weight, respectively (Li et al., 2009).

**TABLE 1:** Overview of studies testing solute diffusion kinetics within bone-cartilage.

References	Technique	Specimen	Tracer	Parameters
Pan et al., 2009	Fluorescence loss induced by photobleaching (FLIP)	Distal femur, C57BL/6J mice, *ex vivo*	Sodium fluorescein 376 Da	Diffusion coefficient
Evans and Quinn, 2005	Confocal microscopy, stage equipped with compression system	Osteochondral cores from bovine humeral heads	TMR 430 Da, Oregon Green 412 Da, AF488 hydrazide 570 Da, Rhodamine Green 10 kDa, Dextran-TMR 10 kDa, Dextran-TMR anionic 10 kDa	Diffusion coefficient
Knothe Tate et al., 1998	Fluorescence microscopy	Metacarpal and tibiae, Sprague–Dawley rats, *ex vivo*	Procion red 300–400 MW, Microperoxidase 1800 MW	Tracer distribution
Ngo et al., 2018	Microtome-blockface episcopic imaging system	Femoral and posterior tibae, Dunkin-Hartley guinea pigs, *ex vivo*	Texas-red 70 kDa, Rhodamine-green 10 kDa	Tracer distribution, cartilage and bone morphology
Serrat et al., 2014	Multiphoton microscopy	Proximal tibia, C57BL/6J mice, *in vivo*	Dextran 10, 40, 70 kDa	Tracer accumulation in growth plate cartilage
Serrat et al., 2009	Multiphoton microscopy	Hindlimbs, Col II/GFP mice, *in vivo*	Fluorescein 332.3 Da	Tracer accumulation in growth plate cartilage
Leddy and Guilak, 2003	Fluorescence recovery after photobleaching (FRAP)	Porcine articular cartilage explants	Dextran 3, 40, 70, 500 kDa	Diffusion coefficient
Shoga et al., 2017	Fluorescence correlation spectroscopy (FCS) and raster image correlation spectroscopy (RICS)	Bovine articular cartilage explants	Dextran 3, 10 kDa	Diffusion coefficient
Li et al., 2009	FRAP	Tibiae, C57BL/6J mice, *in situ*	Sodium fluorescein, Dextran-3, 10 k, Parvalbumin 12 kDa, Ovalbumin 43 kDa	Diffusion coefficient
Arkill and Winlove, 2008	Fluorescence microscopy	Equine cartilage and bone explants	Rhodamine B base (cationic), rhodamine B (neutral polar) Sodium fluorescein Fluorescein (all low MW)	Diffusion coefficient
Pan et al., 2012	FLIP	Knee joints, C57BL/6J mice. Aged, spontaneous OA and DMM, *ex vivo*	Sodium fluorescein 376 Da	Diffusivity

Due to the close association between bone and articular cartilage, studies have also investigated the solute transport at the osteochondral interface. Solute transport was assessed using sodium fluorescein (376 Da) and fluorescence loss induced by photobleaching (FLIP) methods. The results showed a significantly lower (three to four-fold) diffusivity across the osteochondral interface, compared to that of calcified cartilage (Pan et al., 2009). Calculated average diffusion within bone tissue based on previously published results of diffusion with the same tracer in the lacunar-canalicular system, assuming the porosity of the lacunar-canalicular system in the subchondral bone is 5–10% (Wang et al., 2005). By comparing their result with this average diffusivity in subchondral bone, they suggested that calcified cartilage and subchondral bone both have high permeability.

This provides new insight into diffusion at the osteochondral interface however, since these two studies have used different methods for measuring diffusion, a direct comparison is misleading. In addition, calculations and mathematical models proposed by the authors had many assumptions including a homogeneous matrix, the amount of porosity of the lacunar-canalicular system, and the neglect of the other surrounding cells (Pan et al., 2009). Further research is required to compare the diffusion within the subchondral bone and cartilage to that at the osteochondral interface. Overall, the results suggest that solute transport does occur at the osteochondral interface. Electron microscopy of the calcified cartilage matrix also shows non-mineralized regions (∼22% volume fraction), which contains porous channels that may enable solute transport (Pan et al., 2009).

Molecular transport has also been quantified by injecting different size fluorescent tracers in aged guinea pigs, a natural model for OA (Ngo et al., 2018). After a single intracardiac injection of low (10 kDa) and high (70 kDa) molecular weight tracer, the 70 kDa tracer was abundantly detected in the marrow cavity. In contrast, the 10 kDa tracer was detected in meniscus, ligament, and tendon, while none of these tracers were found in articular cartilage. Volumes of tissue containing 10 kDa tracer were significantly lower in older animals compared to younger ones, indicating that molecular transport decreases with age (Ngo et al., 2018).

These studies suggest that tissue sieving properties of the osteochondral interface determine the movement of molecules based on their size. Small molecules can diffuse through the osteochondral interface, however, this transport is altered with age and disease. In contrast, large molecules are unlikely to penetrate some tissues regions in a healthy osteochondral interface, that become available through structural alterations with disease (Ngo et al., 2018). Diffusive transport may also be increased by applying a mechanical load.

### Mechanical Loading Effect on Molecular Transport

Mechanical loading can increase the diffusive transport of molecules across the osteochondral interface. For example, mechanical loading of bone increased the transport and velocity of parvalbumin, a low molecular weight protein (12.3 kDa), which has a size similar to signaling molecules such as sclerostin, in the osteocyte-canaliculi network (Wang et al., 2013).

Molecular transport processes and fluid flow within bone under controlled mechanical loading conditions have been studied on sheep metacarpus using an *ex vivo* perfusion model (Knothe Tate and Knothe, 2000). In this study, before applying mechanical loading, a bolus of tracer was introduced intra-arterially. After loading, the concentration of tracer was significantly higher in loaded bone verses unloaded controls, suggesting that loading can increase molecular transport in bones.

The effect of mechanical loading on transport via the ulna radius interosseous membrane ligament was investigated using different molecular weight fluorescent dextrans. Mechanical loading increased penetration of low molecular weight dextrans (3–500 kDa) through the matrix ligament (Knothe Tate et al., 2012). However, high molecular weight dextrans (2,000 kDa) were only observed in vascular and lymphatic spaces of the bone. They were not detected in the matrix ligament, or in the absence of and after mechanical loading (Knothe Tate et al., 2012). This suggests that although loading increases the magnitude of molecular transport, it cannot overcome the size barrier of the lacunar-canalicular system.

These studies suggest that load-induced fluid flow represents a potential mechanism to increase molecular transport at the osteochondral interface. However, static compression reduces fluid volume and increases charge density of cartilage, resulting in an overall decrease in diffusion, indicating tissue properties also affect diffusive transport of molecules at the osteochondral interface.

### Tissue Properties Effect on Molecular Transport

Under physiological conditions, ECM composition can affect the diffusion of large molecules. This is due to the density and orientation of collagen fibres, whereby higher density and orientation opposite to the direction of solute transport, can reduce solute mobility and diffusivity.

Changes in ECM due to mechanical injury of articular cartilage explants saw increased diffusion of a range of fluorescent solutes, including fluorescein isothiocyanate, dextrans, insulin, chondroitin sulfate and the X-ray contrast agent sodium iodide (Chin et al., 2013). Since chondrocytes were not functional in these explants, the results highlight a role for solute-matrix interactions, independent of the role of cells. Although OA is associated with changes in the gene expression and activity of chondrocytes and bone cells, it is also associated with structural changes at the osteochondral interface, such as loss of proteoglycans, increased subchondral bone thickness, increased vascularization, formation of osteophytes and microcracks.

At early stage OA, high bone turnover causes bone loss and structural changes in the subchondral bone. This is followed by a reduction in bone remodeling, which results in sclerosis of subchondral bone (Bellido et al., 2010). These changes are expected to affect the molecular transport of signaling molecules and crosstalk of these tissues during disease. Interestingly, measuring diffusion at the osteochondral interface and in calcified cartilage of two animal models of mild OA (aging mice and surgical destabilization of the medial meniscus, DMM) showed no significant difference between diffusivity of OA mice and controls (Pan et al., 2009). This might be due to the limitation of the FLIP method for studying large-scale structures. Consequently, improved and non-invasive high-resolution techniques are required to address this issue and track the changes in molecular transport at the osteochondral interface *in vivo*.

## The Use of Contrast Agent Enhanced Imaging Techniques to Study Molecular Transport in Cartilage

Different techniques have been used to study molecular transport at the osteochondral interface, however, their application has been limited to *ex vivo* studies. This includes the use of fluorescence recovery after photobleaching (FRAP) which allows measurement of diffusion of fluorescently labeled molecules. Although FRAP has been be used to study small areas of cartilage or biofabricated scaffolds, it is not feasible for use in large-scale for cartilage tissue due to its complex structure (Nettles et al., 2004). Diffusion cell experiments and solute absorption/desorption techniques measure the transport across the tissue *ex vivo* and cannot be used *in vivo* since the setup relies on optical microscopy. Moreover, they are not accurate for heterogenous tissues, such as articular cartilage, which is comprised of different layers with different structural properties.

Among the methods that have been proposed so far, CT imaging of articular cartilage can be used *in vivo* and at large scale (Joshi et al., 2009; Kulmala et al., 2010; Choi et al., 2019). Contrast agent-based clinical imaging of articular cartilage, such as CT imaging, relies on the transport of contrast agents to and through the cartilage ECM. This method of studying molecular transport can be used as a diagnostic technique to track changes at the osteochondral interface in degenerative diseases, such as OA. It can also provide useful information to design therapeutic molecules and drug delivery systems for these conditions (DiDomenico et al., 2018). Diffusion through the articular surface is the primary route of transport for contrast agents within cartilage (Bashir et al., 1996; Nelson et al., 2018; Bhattarai et al., 2020; Freedman et al., 2020; Meng et al., 2020). A summary of the relevant studies using contrast agent-based diffusion methods for micro-CT is shown in Table 2.

**TABLE 2:** Overview of studies using contrast agent-based diffusion within cartilage.

References	Technique	Specimen type	Contrast agent	Parameters
Kulmala et al., 2010	Peripheral quantitative computed tomography (pQCT)	Articular cartilage discs from bovine patella	Ioxaglate, Gadopentetate, Iodide, Gadodiamide	Diffusion coefficient
Arbabi et al., 2015	MicroCT	Equine osteochondral explants	Iodixanol	Diffusion coefficient
Kokkonen et al., 2017	MicroCT	Articular cartilage discs from bovine patella	Iodine, Gd-DTPA	Partition coefficient and diffusion fluxes
Silvast et al., 2013	pQCT	Articular cartilage discs from bovine patella	Ioxaglate, iodine	Diffusion and partition coefficient
Freedman et al., 2020	MicroCT	Metacarpophalangeal joint from cadaveric hands	Ioxaglate, CA4+	Contrast agent tissue concentration, time to reach equilibrium
Bhattarai et al., 2020	MicroCT	Osteochondral plugs from human cadaver	CA4+, gadoteridol	Contrast agent tissue concentration and partition
Meng et al., 2020	MicroCT	Osteochondral plugs from human tibial plateaus	Ohexol	Contrast agent tissue concentration, diffusion flux and diffusion coefficients
Nelson et al., 2018	MicroCT	Equine osteochondral plugs from the femoral condyles	CA4+	Diffusion time constant and time to reach equilibrium
Kokkonen et al., 2011	MicroCT	Osteochondral plugs from bovine patella	Ioxaglate (Hexabrix), Sodium iodide (NaI)	Diffusion coefficient, diffusion flux

### Diffusion of Contrast Agents Within Cartilage

Measuring the diffusion of four contrast agents, ioxaglate, gadopentetate, iodide and gadodiamide in bovine articular cartilage, showed that the diffusion coefficients of these agents were relatively low (142.8–253.7 m^2^/s). However, diffusion through the articular surface was faster than deep cartilage (Kulmala et al., 2010). In addition, iodide diffuses into cartilage significantly faster than the other three contrast agents in both surface and deep zones, likely due to its atomic size (Kulmala et al., 2010). This study suggests that the diffusion coefficient correlates with cartilage composition, which may be used as a tool for tracking cartilage structural changes.

Differences in transport of solutes across cartilage zones has also been investigated by combining experimental and modeling approaches (Arbabi et al., 2015). Axial diffusion of the neutral solute, iodixanol into cartilage was monitored using calibrated microCT images for up to 48 h. A biphasic-solute computational model was fitted to the experimental data to determine its diffusion coefficients in cartilage. Cartilage was modeled either using one single diffusion coefficient (single-zone model) or using three diffusion coefficients corresponding to superficial, middle, and deep cartilage zones (multizone model). The results showed that the diffusion coefficient of iodixanol in the superficial zone was at least one order of magnitude higher than that of the middle zone. One of the main differences between these zones is their amount of glycosaminoglycan (GAG) content. By having a negative charge, GAG repel the contrast agents with negative charge (inverse correlation) and bind to the ones with positive charge (direct correlation). However, since iodixanol is neutral, GAG content alone cannot explain the large differences between the diffusion coefficients of the different cartilage zones (Arbabi et al., 2015). This finding suggests that diffusion across different zones of the cartilage is affected not only by the charge of solutes and GAG content of cartilage, but also by ECM composition and/or structure, such as water content and the orientation of collagen fibers. Diffusion across cartilage may also be affected by concentration of the contrast agent.

Alteration of ECM structure and solute-matrix interactions due to cartilage injuries additionally affects solute transport through cartilage (Kokkonen et al., 2011; Chin et al., 2013). Effects of mechanical injury on transport of negatively charged contrast agents in cartilage has been investigated (Kokkonen et al., 2017). Using cartilage plugs injured by mechanical compression protocol, effective partition coefficients and diffusion fluxes of different contrast agents were measured using high resolution microCT imaging. For all contrast agents studied (Sodium iodide, sodium diatrizoate and Gd-DTPA) effective diffusion fluxes increased significantly, particularly at early time points of the diffusion process. Moreover, the results suggest that alterations in contrast agent diffusion flux provides a more sensitive indicator for assessment of cartilage matrix integrity than partition coefficient and the equilibrium distribution of solute.

### Effect of Concentration on the Transport of Contrast Agents in Cartilage

The effect of concentration on diffusion of anionic contrast agents like ioxaglate and iodide in cartilage have been investigated *in vitro* (Silvast et al., 2013). Samples were imaged with a clinical peripheral quantitative CT scanner before immersion in contrast agent, and after several time points in the diffusion and partition coefficients of these contrast agents were not affected by concentration at the equilibrium. One possible explanation given by authors for these results is that dependency of diffusion to concentration is only minor in diluted solutions (Silvast et al., 2013). Changes in contrast agent diffusion reflect the changes in composition and structure of articular cartilage (Leddy and Guilak, 2003; Evans and Quinn, 2005; Arkill and Winlove, 2008). However, clinical use of contrast agents for this purpose, requires highly consistent concentration before their administration.

Although these *ex vivo* microCT studies provide insights into the diffusion transport of contrast agents through articular cartilage, it is unknown whether the same mechanisms are relevant in *in vivo* transport of molecules at the osteochondral interface. *In vivo* experiments would enable time-lapse study of changes, and correlation of these changes with OA progression.

## Conclusion and Perspectives

The osteochondral interface plays a critical role in joint function and disease by connecting joint compartments and allowing the exchange of signaling and nutritional molecules between them. Chondrocytes and osteocytes sense and respond to chemical and mechanical stimuli by releasing the signaling molecules that they produce. The exchange of molecules between these two tissues provides an integrated response to environmental stimuli affecting joint homeostasis.

Studies using a novel imaging method based on FLIP have shown intravenous administration of fluorescent molecules can diffuse through the bone-cartilage interface (Knothe Tate et al., 1998; Pan et al., 2009). Using this method, transport capacity was higher in mouse models of OA than normal joints (Pan et al., 2012), however, this approach is not compatible with time-lapse measurement. Capturing transport variations over time is the next step toward expanding the knowledge of OA pathways. Coupling mechanical tests and microCT longitudinal measurement approach may enable measurement of joint remodeling responses at both organ and tissue levels. It may also provide insights into the association between molecular transport alteration and joint disease progress under mechanical loading.

Currently, there is no single preclinical imaging modality available for imaging molecular transport and its correlation with the structural changes of the osteochondral interface at the onset and during the progression of joint diseases, such as OA. Capturing *in vivo* molecular transport over time in the osteochondral interface is the next step toward expanding the knowledge of OA nutrient pathways.

To achieve this goal, a non-toxic contrast agent for high-resolution molecular transport imaging is desirable for imaging *in vivo* molecular transport changes in the osteochondral interface. MicroCT is used for capturing high resolution structural details of cartilage and bone (Stok et al., 2016). By using a non-toxic contrast agent capable of diffusing through the osteochondral interface, it could be possible to track *in vivo* molecular transport and structural changes of the tissues. The fundamental knowledge provided by this method may be beneficial for designing advance drug delivery systems for joint disorders. This method could be also be used for time-course studies, at the research level, minimizing the number of animals used per study, and providing improved controls for interpreting results. In addition, this method would be non-invasive, and a promising diagnostic tool to track structural changes of cartilage with the progress of disease in subjects, in a longitudinal manner.

## Author Contributions

KS and MO conceived and designed the review. MO drafted the manuscript. All authors contributed to the manuscript and critically reviewed the manuscript prior to submission.

## Conflict of Interest

The authors declare that the research was conducted in the absence of any commercial or financial relationships that could be construed as a potential conflict of interest.
